# Energy harvesting for the implantable biomedical devices: issues and challenges

**DOI:** 10.1186/1475-925X-13-79

**Published:** 2014-06-20

**Authors:** Mahammad A Hannan, Saad Mutashar, Salina A Samad, Aini Hussain

**Affiliations:** 1 Department of Electrical, Electronic & Systems Engineering, Faculty of Engineering and Built Environment, UniversitiKebangsaan Malaysia, 43600 UKM, Bangi, Selangor, Malaysia; 2 Department of Electrical and Electronic Engineering, University of Technology, Iraq, Baghdad

**Keywords:** Energy harvesting, Implantable biomedical devices, Electromagnetic, Human motion, Kinetic energy, Inductive coupling link, Piezoelectric material

## Abstract

The development of implanted devices is essential because of their direct effect on the lives and safety of humanity. This paper presents the current issues and challenges related to all methods used to harvest energy for implantable biomedical devices. The advantages, disadvantages, and future trends of each method are discussed. The concept of harvesting energy from environmental sources and human body motion for implantable devices has gained a new relevance. In this review, the harvesting kinetic, electromagnetic, thermal and infrared radiant energies are discussed. Current issues and challenges related to the typical applications of these methods for energy harvesting are illustrated. Suggestions and discussion of the progress of research on implantable devices are also provided. This review is expected to increase research efforts to develop the battery-less implantable devices with reduced over hole size, low power, high efficiency, high data rate, and improved reliability and feasibility. Based on current literature, we believe that the inductive coupling link is the suitable method to be used to power the battery-less devices. Therefore, in this study, the power efficiency of the inductive coupling method is validated by MATLAB based on suggested values. By further researching and improvements, in the future the implantable and portable medical devices are expected to be free of batteries.

## Introduction

Energy harvesting devices generate electric energy from their surroundings through direct energy conversion [1]. To date, implantable biomedical devices are powered using a couple of wires; this setting may cause skin infections, discomfort, and other hazards to patients. Currently, implanted batteries provide the energy for implantable biomedical devices. However, batteries have fixed energy density, limited lifetime, chemical side effects, and large size. Thus, researchers have developed several methods to harvest energy for implantable devices. Devices powered by harvested energy have longer lifetime and provide more comfort and safety than conventional devices. A good solution to energy problems in wireless sensors is to scavenge energy from the ambient environment. Energies that may be scavenged include infrared radiant energy, thermal energy (solar–thermal, geothermal gradients of temperature, combustion), kinetic energy (wind, waves, gravity, vibration, and body motion), wireless transfer energy, and RF radiation energy (inductive and capacitive coupling).

The energy harvesting from human or environmental sources has been provided to be an effective alternative. Many researchers have found solutions may be useful and incorporated as a review paper. Therefore, several studies provide reviews which focus only on one type of classified energy harvesting for biomedical implanted devices such as kinetic energy from body motion, vibration, piezoelectric material, [2-4] or wireless transfer energy, [5], or using thermal and solar energy from the environment sources as given in [6-8]. In 2010, the researchers Paulo & Gaspar [9] and in 2011, Jaeseok *et al.*[10] they provided a good study of power harvester using the human body motion to power the biomedical sensor nodes. This review provides a detailed investigation of the literature concerning the energy harvesting for biomedical implanted. The purpose of this review is to discuss and classify all the types of the energy harvesting used in wireless telemetry bio-devices and biomedical implanted devices to provide a good background on the challenges and problems that are being faced and to develop appropriate solutions.

## Methods

**Figure 1:**
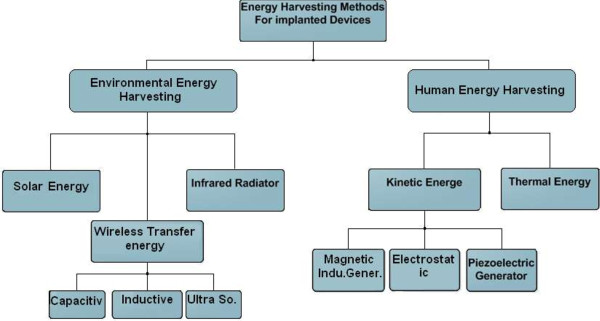
Energy harvesting methods used in biomedical applications.

Implantable biomedical devices may be classified into two types. The first type includes devices powered by energy harvested from the human body and covered by secondary forest. The second method includes those powered by energy harvested from the environment and covered by secondary forest. All types of energy harvesting methods used in biomedical applications are presented in Figure 1, and as follows.

### Human energy harvesting

Human activities are sources of kinetic and thermal energies. Different body activities produce different levels of power. Sleeping can produce approximately 81 mW of power, whereas sprint walking and motion produce 1630 mW of power [9]. The human body can retain temperature even when the ambient temperature changes. This property maintains the metabolic processes necessary for energy production even if the surrounding temperature is extremely cold. Therefore, the two types of energy generated by human body activities such as kinetic and thermal energy harvesting are investigated in this section.

### Kinetic energy

Human body activities are a promising source of energy for implantable biomedical devices. Kinetic energy is a readily available energy source for both human and environment energy harvesting devices. This section briefly explains the principles of different transducers for obtaining electrical energy from kinetic energy, including piezoelectric, magnetic induction generator, and electrostatic transduction methods [11-13] and as follows.

### Piezoelectricity

**Figure 2:**
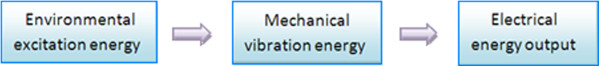
The energy conversion flowchart.

**Figure 3:**
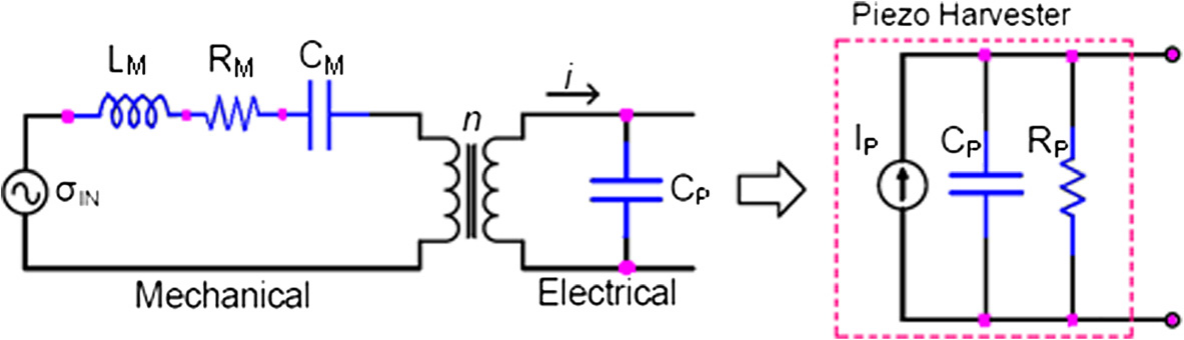
Equivalent scheme of the piezoelectricity converter.

The first piezoelectric effect was discovered by brothers Jacque and Pierre Curie in 1880. They found that certain materials, when subjected to mechanical strain, suffer an electrical polarization that is proportional to the applied strain. This piezoelectric effect is used to convert mechanical motion to electrical energy. The flowchart of this conversion is observed in Figure 2, and the equivalent scheme is shown in Figure 3.

**Figure 4:**
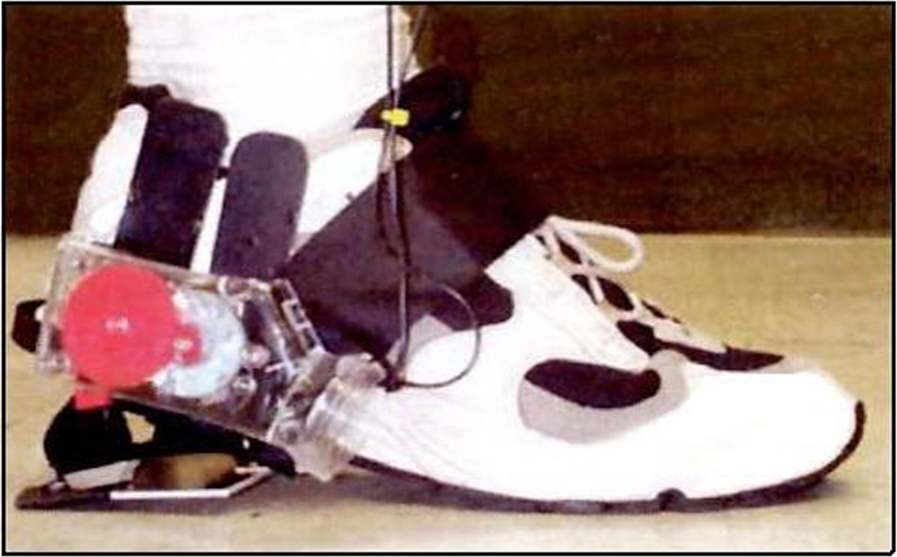
Magnetic generator adapted in a shoe.

In 1990, the Media Lab at Massachusetts Institute of Technology (MIT) fabricated the first energy harvesting device based on human walking; this device was used to convert human motion into energy for wearable electronic applications. In 1998, Paradiso *et al*. [14] implemented a spring magnetic generator in the shoe heel to produce approximately 1 W of power (Figure 4). Despite its capacity to generate power, this prototype causes discomfort and can be applied only to patients who can walk normally.

In 2001, Paradiso *et al.*[15] again integrated piezoelectric elements in two positions in the shoes: one on the heel and the second on toes. This prototype generates 8.3 mW and 1.3 mW, respectively. However, this prototype has limited power generation capacity and can be applied only to patients who can walk normally. To improve the results obtained in [14,15], Kornbluh compressed piezoelectric elements in the heel of a boot [16]. Although, the generated power given by [16] is improved, but this prototype is limited by the same problems stated in [14,15]. Piezoelectric materials generate electrical energy when exposed to mechanical pressure. Ramsay and Clark [17] used a square PZT-5A to generate energy with a maximum power of 2.3 μW from the typical fluctuations of blood pressure. Sohn *et al.*[18] investigated the use of circular and square polyvinylidene fluoride plates to harvest energy from blood pressure fluctuations. Platt *et al.*[19] embedded piezoelectric ceramics within orthopedic implants to generate 4.8 mW. Hong *et al.*[20] embedded piezoelectric ceramics within knee replacement implants to generate 1.2 mW. In 2010, Shaban *et al.* embedded four piezoelectric ceramic plates within knee replacement implants to generate 1.81 mW [21]. The principal problem of the aforementioned method is the size and thickness of the plates.

### Electrostatic energy

**Figure 5:**
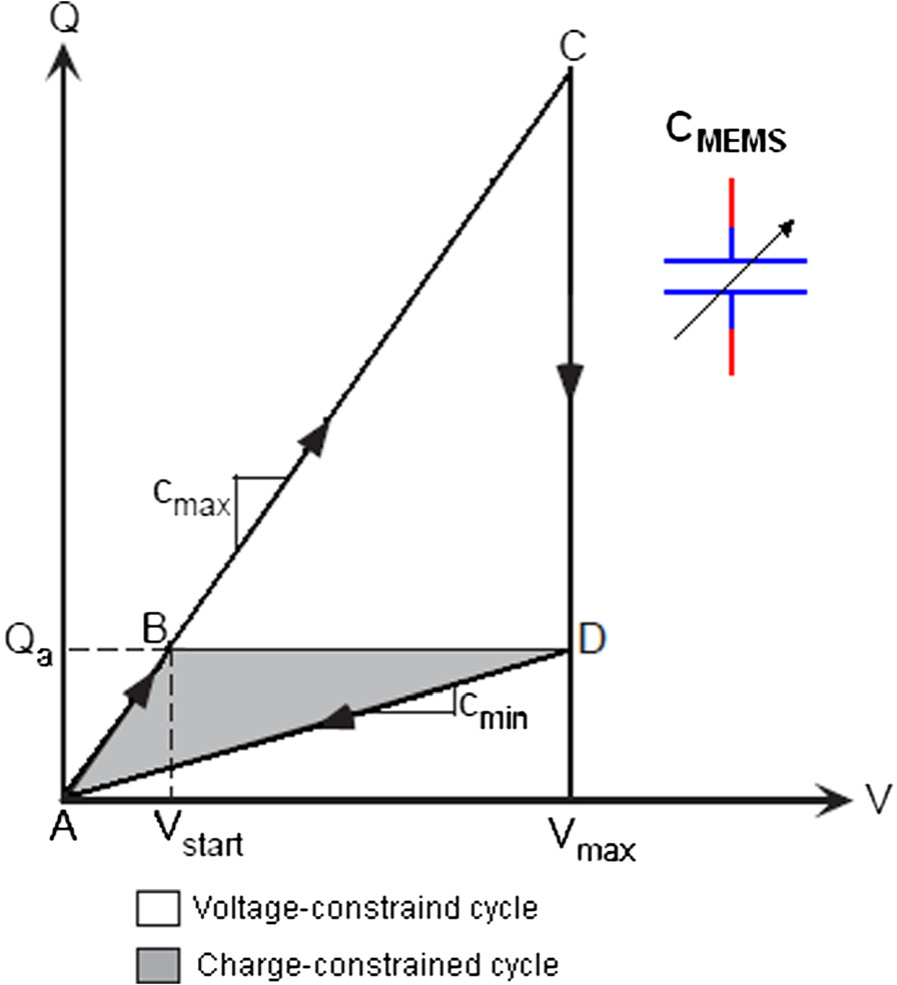
Diagram explaining electrostatic energy conversion.

Electrostatic generators produce electricity via electrostatic induction. These devices convert mechanical vibration into electrical energy by moving part of the transducer versus an electrical field. The conversion has two possibilities: with fixed charge or fixed voltage. Figure 5 shows the charging and discharging processes of the capacitance following a constant charge path (A-B-D-A) or a constant voltage path (A-C-D-A) [22]. This technique is suitable for micro-implanted devices (e.g., implantable biosensors) operated with low power.

**Figure 6:**
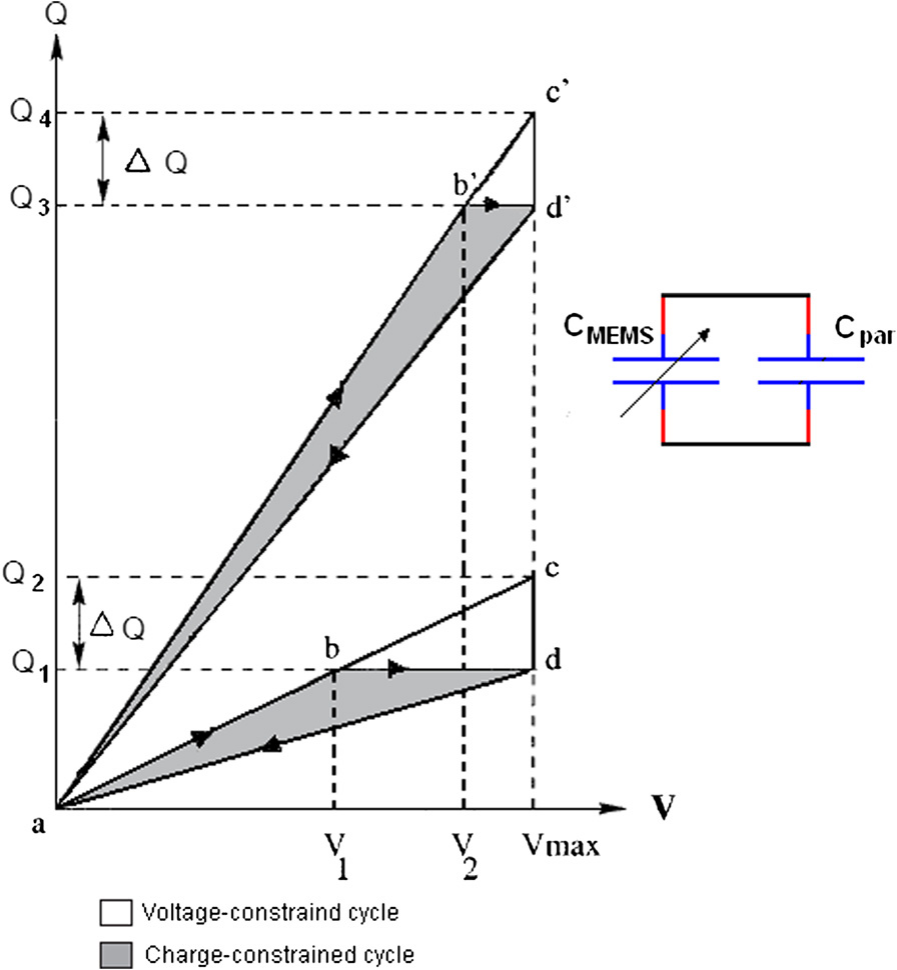
The developed electrostatic energy conversion.

Meninger *et al.*[23] developed the condition given in [22] and produced an electrostatic generator that utilizes a variable micro-machined capacitor. They added the parallel capacitor C_par_ to the microelectromechanical systems (MEMS) as shown in Figure 6. This system can generate 8 μW of power. However, the added capacitor may increase the initial charge; therefore, it must be set carefully to the desired value.

Kinetic generators based on electrostatic transducers use variable capacitors. The position of the plates can be changed by external force, and these generators can operate under fixed charge or fixed voltage. Under a fixed charge, the external force changes the voltage across the capacitor; under a fixed potential, plate movement generates current through the capacitor. Independent of the operation mode, kinetic generators usually have to be recharged to operate. These generators have low efficiency when high power is required but work well with devices that have low power requirements, such as implantable biosensors.

In 2000, Tashiro *et al.*[24] proposed an electrostatic generator (ESG) to provide 58 μW when placed in motion by a force simulating the cardiac signal, taking advantage of a MEMS capacitor with variable capacitance ranging from 32 nF to 200 nF. In 2002, the same team tested their proposed ESG experimentally in an animal and obtained a heart rate of 180 bpm [25]. Miao et al. [26] proposed a non-resonant MEMS electrostatic generator for biomedical applications and produced 80 μW when motivated with a speed of up of 10 m/s. This generator operates over a wide range of oscillation frequencies with a constant charge. A piezoelectric generator was proposed based on lead zirconate titanate to generate 40 μW [27]. Another piezoelectric generator was proposed based on aluminum nitride with an unpackaged device to provide 60 μW [28]. Different types of commercial electrostatic generators are currently being widely used in different applications [29].

### Magnetic induction generator

**Figure 7:**
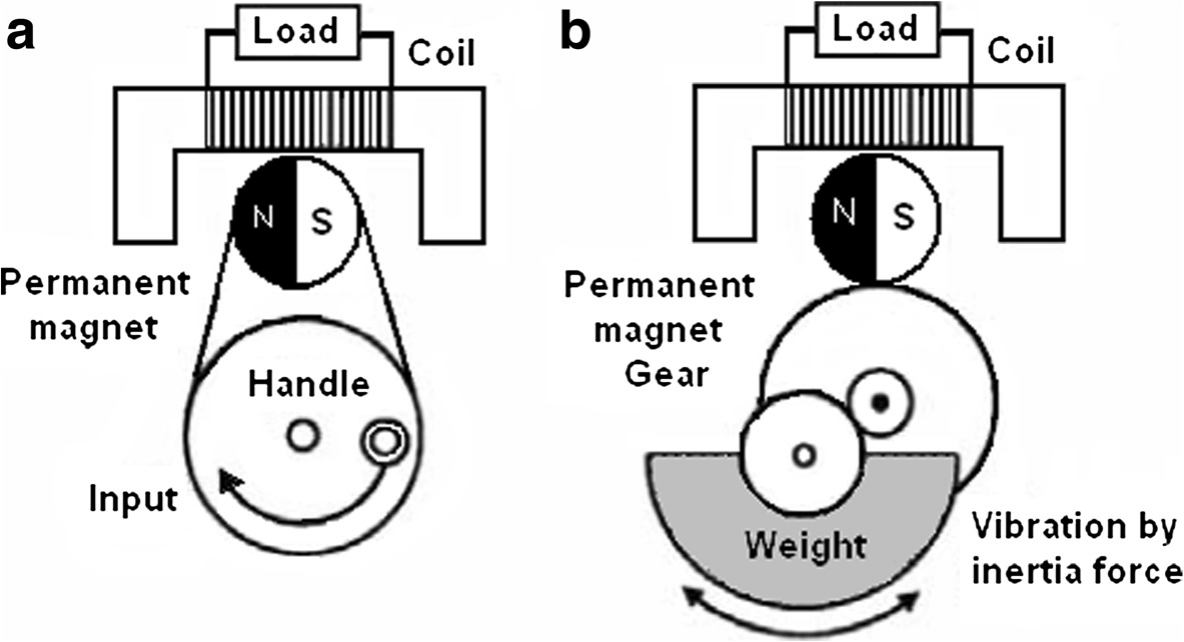
The mechanical generators Types: a) relative movement, b) rigid body.

Mechanical generators that produce electromagnetic energy have two types. The first type uses relative motion where the generating system is fixed, and the second type uses rigid body motion where the inertia force of the weight is installed on the generator. Figure 7 shows the basic setting of these generators. Hosaka [30] investigated both types by using bicycle generators, mobile phones, and radios; he concluded that the second type is more vulnerable to vibratory movements than to constant movements because it uses inertia, that is, the resistance to movement. Electromagnetic transducers can induce flux changes by rotating the circuit along an axis, thereby changing the surface associated with the magnetic flux. A previous study [31] used this method to power the quartz wristwatch. This “Seiko Kinetic” approach has been successfully tested in biomedical applications; it can utilize heartbeats to charge the implanted pacemaker battery [32].

**Figure 8:**
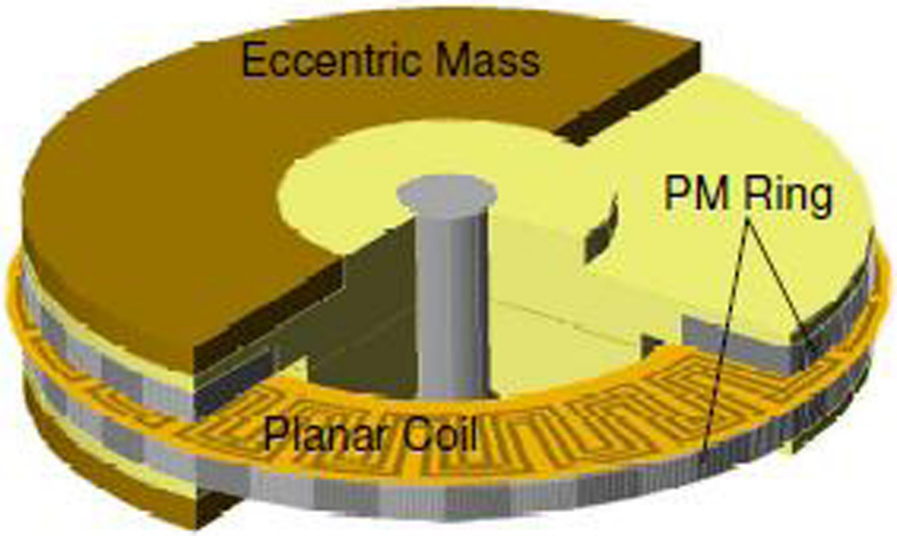
The 3D schematic of the axial flux generator.

Using human walking, Amirtharajah *et al.*[33] in 1998 used electromagnetic VDRG built to generate 400 μW of power. A new electromagnetic MEMS VDRG was fabricated by Li *et al*. [34] to generate 10 μW of power at 2 V DC using 64 Hz of input frequency, the limitation of the generated energy is the issue [34]. In 2001, Williams *et al.*[35] used the same prototype given in [34] to generate 0.3 μW of power from a 4 MHz excitation input. In 2009, an axial flux generator to generate energy by electromagnetic indication on the planar coil is produced by Edward as shown in Figure 8[36]. This generator involves a gear-shaped, planar coil and a ring-attached eccentric weight. This device based on rigid body was fixed on the ankle and, during walking, is used to provide 3.9 μW of power to diminutive biomedical devices.

### Thermal energy

**Figure 9:**
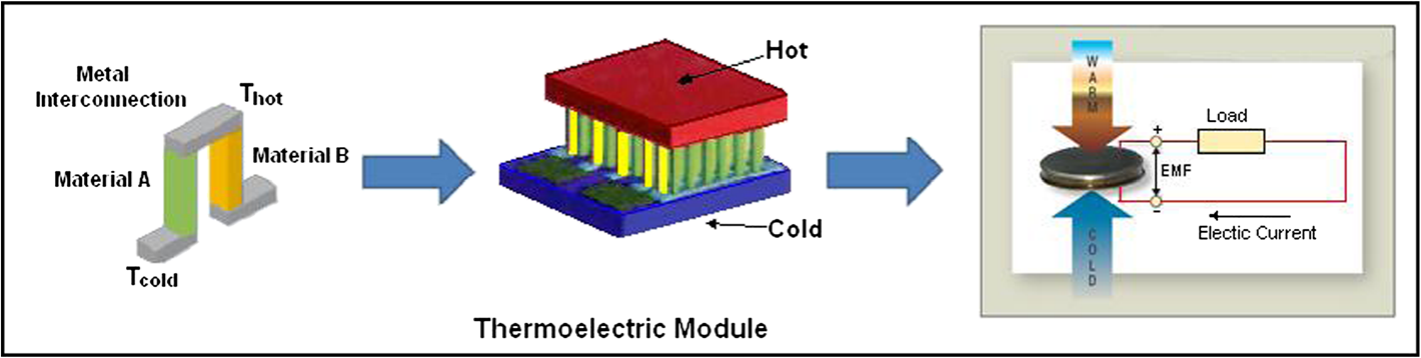
Thermoelectric module with thermopile and equivalent scheme.

Thermo-electric harvester technologies are promising ways to produce minimal power from the temperature differences (Seebeck effect). This power is sufficient for bio-implantable devices, such as implanted nerve and muscle stimulators, cochlear hearing replacements, and wireless patient diagnostics. A thermoelectric generator contains a large number of thermocouples connected electrically in series with high thermal resistance and thermally in parallel forms a thermopile as shown in Figure 9. This structure is ideal for harvesting energy from the body and dissipation heat. However, Carnot efficiency [η_c_ = (Th–Tc)/Th] limits the percentage of energy extracted by the generator. For example, η_c_ is 1.6% at room temperature when the temperature gradient is 5K, and the best thermoelectric materials achieve maximum Carnot efficiency values of up to approximately 17% for small temperature gradients [37]. In 1999, Stevens [38] investigated a standard thermoelectric material where η_c_ ranges from 0.2% to 0.8% and leads to overall conversion efficiencies for temperature differences from limited 5K to 20K.

The thermocouple involves p-type (“A” material) and n-type (“B” material) semiconductors with positive and negative Seebeck coefficients, respectively. The voltage generated across the thermocouple because of the difference between hot and cold junctions (T_h_-T_c_) can be expressed as

(1) $$V=\int_{T_{h}}^{T_{c}}\left[S_{B}\left(T\right)-S_{A}\left(T\right)\right]\mathrm{dT}$$

where S_A_ and S_B_ represent the Seebeck coefficients of the two materials. Thus, an electrical proportional current flows through the electrical load, which is connected in series with the thermo-electric generator.

The literature offers several examples of thermopiles exploiting human heat. Significant efforts have been exerted to improve the technology performance of these systems. However, the power range of thermoelectric harvesters when exploiting human heat is low and typically does not exceed a few hundred microwatts when a thermal difference below 5K is applied. Stark and Stordeur [39] obtained 1.5 μW with a 0.19 cm device exploiting a thermal gradient of 5K. Similar results were obtained by Strasser *et al.*[40] with a device that can offer 1 μW with an area of 1 cm and a thermal gradient of 5K. The limited energy in [39,40] are the main issue. A commercially available solution, which is proposed by Thermo [41], involves a device that can produce up to 30 μW (10 A with a voltage drop of 3 V) when a temperature difference of 5K is applied. This device has a volume of 95 mm and a weight of 0.23 g.

### Environmental energy harvesting

Environmental bio-energy harvesting (EEH) is a means of powering biomedical devices by scavenging many low grade ambient energy sources such as infrared, solar and wireless energy transmission, and their conversion into useable electrical energy to power the implanted devices. EEH devices are therefore potentially attractive as replacements for implanted batteries. They also hold the promise of one day enabling the powering of a range of implantable and wearable medical devices.

### Infrared radiation

Infrared radiation is an energy harvesting method that powers large bio-implantable devices (e.g., cardiac and brain pacemakers) by exploiting an external infrared source. This method suffers has several drawbacks, such as large size, relatively low harvested energy, high power consumption, and skin heating. The implanted photodiode array is the principal element in infrared radiation devices [42]. Goto *et al.*[43] produced a device that can be used in implantable cardiac pacemakers; this device can transmute 4 mW of power when the device is powered with 2.8 DC voltages. The transmitted power increases the skin temperature by 1.4°C, which may increase skin temperature and damage soft tissue.

### Solar energy harvesting

A solar energy harvester is a mature technology motivated by natural photosynthesis using dye-sensitized solar cells or Gräetzel cells [44]. Although this technology is not yet applicable to bio-implantable devices, it is expected to be one of the key technologies in biomedical applications for subcutaneous implanted devices. Most solar cells are made from semiconductor materials, which consist of 89% of crystalline silicon, 10% of amorphous silicon, 0.5% of cadmium telluride, dieseline, copper indium, and gallium arsenide. The structure of this device involves an anode and a cathode, between which a molecular dye exists and converts solar light into electrons. These electrons reach the anode electrode by a stratum of titanium dioxide, and then the electron holes generated into the dye reach the cathode electrode through a liquid electrolyte. The conversion of light to the electrons may be useful for biomedical devices.

### Wireless transfer energy

For the implantable devices applications, currently, the wireless transfer energy consider as the robust method that can be used to power implanted devices instead of batteries. There are three main methods, which we described in details and as follows.

### Ultrasonic energy harvesting

**Figure 10:**
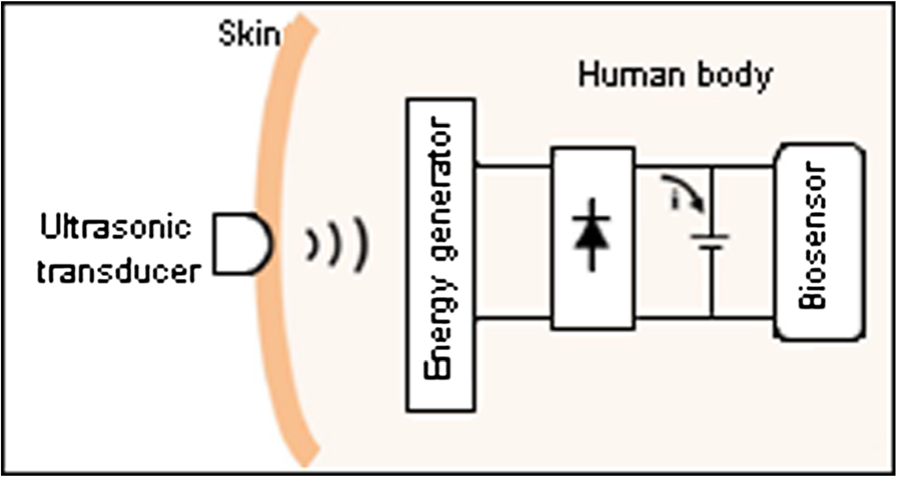
The ultrasonic transmission method with implanted device.

Ultrasonic transmission is a modern method of energy harvesting. This method is relatively safe for the human body and does not cause electronic interference with other electromagnetic devices [45]. In 2004, Phillips *et al.*[46] designed a device that allows pulsed ultrasound to provide a milliamp order of currents in piezoelectric devices. Tower *et al.*[47] produced a device that may be suitable for potential monitoring. This device converts the energy of a surface-applied ultrasound beam to a high-frequency current. Figure 10 shows the ultrasonic transmission method where the ultrasonic generator is fixed on the skin and coupled energy with the biosensor. The MEMS inside the body absorbs the ultrasound energy and converts it into electrical charge. The design is considerably higher than 100 pW of the 2-D electrostatic power harvester reported by Bartsch *et al.*[48]. In 2010, Zhu *et al.*[49] exploited ultrasonic waves to power implanted biosensors with 21.4 nW, which is higher than the power reported in [48]. This novel ultrasonic generator was designed based on two degree-of-freedom (2-DOF) MEMS. In general, this method is under improvement to overcome disadvantages such as relatively low harvested energy and large size caused by MEMS devices.

### Capacitive coupling link

**Figure 11:**
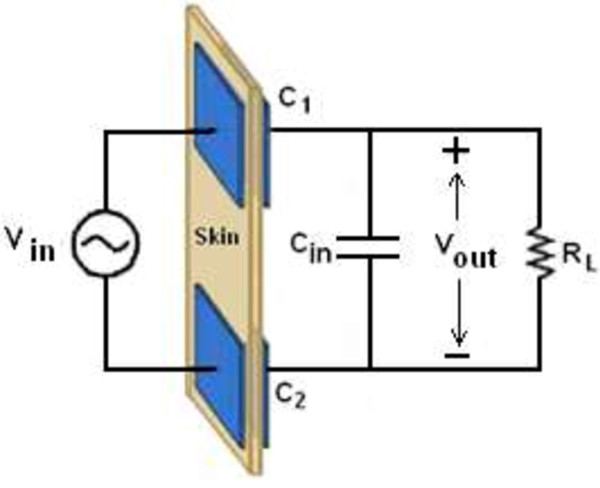
The simplified capacitive coupling link.

The capacitive coupling link approach is used to transfer data and power to the implanted devices at short wireless communications. The principle behind this approach is based on two parallel aligned plates that behave as capacitors. The first plate is fixed outside the body and attached to the skin; the second plate is implanted inside the body and connected to the implanted device as shown in Figure 11. In 2006, Culurciello and Andreou [50] used this approach to transfer power to the implantable devices. Canegallo *et al.*[51] and Fazzi *et al.*[52] used this approach in 2007 and 2008 to transfer data, respectively. Again, Sodagar *et al.* used this approach to transfer power and data to the implanted micro-system as a new application [53]. The capacitive coupling link uses the electric field as a carrier to transfer data and power through the skin, which acts as a dielectric separated between the two plates.

Referring to Figure 11, we analyzed the voltage transfer rate as follows: *V*_*in*_ is the input voltage, *C*_*1*_ and *C*_*2*_ are the capacitances between the implanted and external plates, *C*_*in*_ is the equivalent input capacitance of the implanted circuits, and *R*_*L*_ is the equivalent “ac” resistance of the loading network. The equivalent capacitors *Ceq* is given in

(2) $$C_{\mathrm{eq}}=C_{1}+C_{2}$$

Assuming *Cin* < <*Ceq*, then

(3) $$V_{\mathrm{out}}=V_{\mathrm{in}}\left[\frac{R_{L}^{2}}{R_{L}^{2}+X_{C_{\mathrm{eq}}}^{2}}+j\frac{R_{L}X_{C_{\mathrm{eq}}}}{R_{L}^{2}+X_{C_{\mathrm{eq}}}^{2}}\right]$$

and the voltage transfer rate is given by

(4) $$\left|\frac{V_{\mathrm{out}}}{V_{\mathrm{in}}}\right|={\left(\frac{R_{L}^{2}}{R_{L}^{2}+X_{C_{\mathrm{eq}}}^{2}}\right)}^{\frac{1}{2}}$$

Thus, *V*_*out*_ is maximized when *X*_*Ceq*_ < <*R*_*L*_.

The major disadvantage of this method is that the plates may increase the tissue temperature, causing patient discomfort. In addition, the human body is a non-magnetic material. Negligible magnetic field losses indicate that human tissue absorbs the electric field [54].

### Inductive energy harvesting

**Figure 12:**
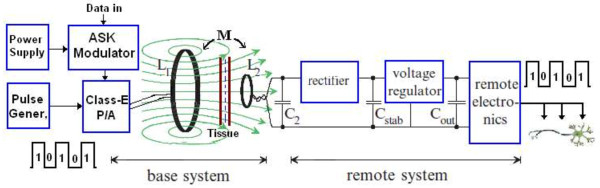
Block diagram of an inductive coupling link system.

Now days, inductive coupling link is an attractive developing technology for biomedical applications in short communication. Therefore, in this section, a deep survey is done, and simulation and validation based on suggested values is presented for efficient power transmission. This technology uses magnetic coupling as the communication environment, which is common with radio frequency identification techniques [55,56]. Most studies related to inductive links used frequencies lower than 20 MHz [57,58] to avoid tissue heating caused by power absorption within tissue. Practically, the RF short-range communication transmits low power (less than a milliwatt) and radiates RF power signal from the reader coil antenna, which is mostly designed to offer fixed sinusoidal carrier amplitude, which provides a stable wireless transfer power. The stability of the RF signal provides a high readability for DC voltage at the implant device in terms of the distances from the reader coil. Figure 12 shows a schematic of an inductive link performing unidirectional data transmission for the bio-implanted micro system.

**Figure 13:**
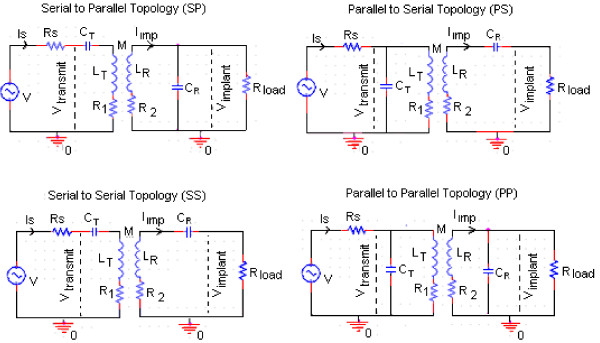
The four possible resonance circuits in inductive coupling.

The biodevice system is composed of two coils. One is located inside the human body (implant), and the other is located outside the body (reader). The connections in a passive system have four resonance possibilities: serial-to-parallel topology (SP), parallel-to-serial topology (PS), serial-to-serial topology (SS) and parallel-to-parallel topology (PP) as demonstrated in Figure 13, respectively. To ensure better power transfer efficiency of the inductive coupling link, both sides of the link are tuned at the same resonant frequency *f*_*o*_*.* In most cases, the primary circuit (reader) is tuned in series resonance to provide a low impedance load for driving the transmitter coil, where the secondary circuit (implant) is almost invariably parallel, and uses an LC circuit to drive a nonlinear rectifier load.

In practice, the number of the coil turns can be changed based on wire properties and coil shape. A more practical approach involves measuring the inductance during construction and odd turns until the specified inductance is reached. However, measuring inductance accurately requires a highly specialized and expensive inductance meter [59]. Practically, the resonance frequency *f*_*0*_ can be calculated by using Equation (5) [60,61]. Numerous formulas can be used to estimate the number of turns required to achieve a particular inductance *L*. For example, the equations in Table 1 provides the (N) turns based on loop radius (a), loop height (h), loop width (b), (d) wire diameter (R), radius of the loop coil (r), and magnetic inductance *L*. However, these equations can only provide approximations for ideal conditions [62-65].

(5) $$f_{0}=\frac{1}{2\pi \sqrt{\mathrm{LC}}}$$

**Table 1:** Formulas approximate for how many turns are required to achieve a specified inductance

**Formulas**	**References**
$L=N^{2}R{µ}_{0}{µ}_{r}\left[\ln\left(\frac{8R}{a}\right)-0.2\right]$	[62]
$L=\frac{r^{2}N^{2}}{\left(2r+2.8d\right)*10^{3}}$	[63]
$L=\frac{0.31{\left(\mathrm{aN}\right)}^{2}}{6a+9h+10b}$	[64]
$L=2.9\ln\left(\frac{9}{D}-K\right)N^{1.9}$	[65]

Other parameters that should be considered during inductive coupling design are mutual inductance (M) and coupling coefficient with respect to *L*_*T*_ and *L*_*R*_, as proposed by [65]

(6) $$K=\frac{M}{\sqrt{L_{T}L_{R}}}$$

With the SP topology given in Figure 13, the resistor *R*_*1*_ is a combination of effective series resistance of *L*_*T*_, which presents the transmitted coil losses and the output resistance of the power amplifier, whereas *R*_*2*_ is the effective series resistance of *L*_*R*_ as given by (Liu *et al.* 2005) [66] and (Harrison, 2007) [67].The capacitors *C*_*T*_ and *C*_*R*_*are* used to create a resonance on both sides of the link. The resonance frequency (ω_o_) for an *LC* tank for both sides can be calculated as given in (7).

(7) $$\omega_{0}=\frac{1}{\sqrt{L_{T}C_{T}}}=\frac{1}{\sqrt{L_{R}C_{R}}}$$

The quality factor (Q) for the primary and secondary coils is presented in (8) respectively.

(8) $$Q_{1}=\frac{\omega L_{T}}{R_{1}}\mathrm{and}\;Q_{2}=\frac{\omega L_{R}}{R_{2}}$$

For high efficiencies, the efficiency for both side of link should be maximized and this can be occurs when.

(9) $$1<<K^{2}Q_{1}Q_{2}=\frac{K^{2}L_{T}}{L_{R}}*\frac{1}{R_{2}C_{T}}$$

**Figure 14:**
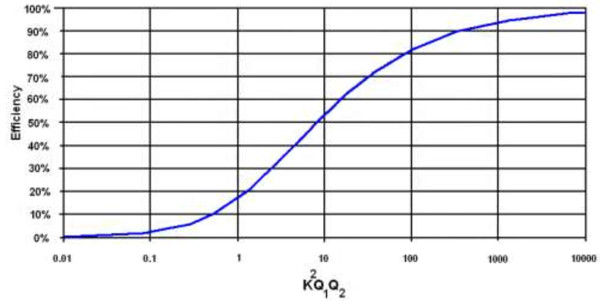
**Maximum achievable link efficiency as a function of (K**^**2**^**Q**_**1**_**Q**_**2**_**).**

Figure 14 shows the total efficiency as a function of (K^2^Q_1_Q_2_), where the efficiency increases with increasing coupling coefficient and quality factor as given in (10) [68,69].

(10) $$\eta_{\max}=\frac{K^{2}Q_{1}Q_{2}}{{\left(1+\sqrt{1+K^{2}Q_{1}Q_{2}}\right)}^{2}}$$

**Figure 15:**
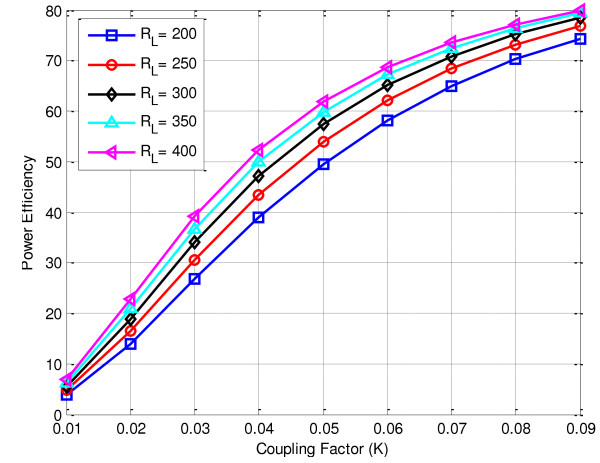
Inductive coupling link against load resistance.

Another factor, which directly affects the total efficiency, is the resistance of the implanted devices (loaded case). For inductive link validation we assumed that the implanted resistance varies between 200 Ω and 400 Ω, resonance frequency ω = 13.56 MHz, external coil resistance *R*_*1*_ = 2.2 Ω implanted coil resistance *R*_*2*_ = 1.6 Ω the coefficient factor is *K* = 0.087, hence, the quality factors for the external and implanted coils are *Q*_*1*_ = 190, *Q*_*2*_ = 53, Ω, respectively. According to (11) [70] the total power efficiency is also increases proportionally with increasing load, and varied between 74% - 80% depending on the proposed implanted resistance as shows in Figure 15.

(11) $$\eta_{\mathrm{total}}=\eta_{T}\eta_{R}=\frac{K^{2}Q_{1}Q_{2}^{3}R_{\mathrm{LR}}R_{\mathrm{load}}}{\left(K^{2}Q_{1}Q_{2}^{3}R_{\mathrm{LR}}R_{\mathrm{load}}+K^{2}Q_{1}Q_{2}R_{\mathrm{load}}^{2}+Q_{2}^{4}R_{\mathrm{LR}}^{2}+2Q_{2}^{2}R_{\mathrm{LR}}R_{\mathrm{load}}+R_{\mathrm{load}}^{2}\right)}$$

## Discussions

The most important issues in designing implantable devices are comfort and safety of patients. Hannan *et al.*[71] reviewed implanted devices and their power consumption, data rate, size, applications, and modulation technique. Power sources and energy harvesting for implanted devices still have to be discussed in detail. Therefore, in this review, we reported all of the methods that can be used to power the implantable devices. Table 2 mentions the energy harvester methods, generated power, and their advantages and disadvantages. The wireless harvesting energy using inductive coupling link is the best and most suitable method to power subcutaneous implanted devices with low bands of industrial, scientific, and medical (ISM) frequencies. However, most of the methods (e.g., implantable batteries and capacitive methods) are still under investigation and provide low energy. They may also cause hazards and skin infections. The kinetic method suffers from relatively large size and low power. Thermal and ultrasound methods produce extremely low energy, which is not suitable for certain implantable devices. However, these methods may be suitable for implantable bio-sensors in the future.

**Table 2:** Comparison between the energy harvesting methods and there challenges

**Energy harvesting method**	**Technique**		**Reference**	**Generated power**	**Advantage**	**Disadvantage**
**Human energy harvesting**	Kinetic energy	Piezoelectricity	[14]	1 W	- No separate voltage source	- Difficult to integrated with Micro-system
			[15]	8.3 & 1.3 mW	-Voltage of 2–12 volts	
			[17]	2.3 mW		
			[19]	4.8 mW	-No mechanical stops	
			[20]	1.2 mW		
			[21]	1.81 mW	-Highest energy density	
		Electrostatic generator	[23]	8 μW	-Easier to integrate with electronics and micro-systems	- Separate voltage source needed
			[24]	58 μW		
			[26]	80 μW		
			[27]	40 μW	-Voltage of 2–12 volts	- Mechanical stops needed
			[28]	60 μW		
		Magnetic induction generator	[33]	400 μW	No separate voltage source	-Maximum voltage is 0.1 volts
			[34]	10 μW	No mechanical stops	-Difficult to integrate with micro-systems
			[35]	0.3 μW		
			[36]	3.9 μW		-Energy generated only during walking
	Thermal energy		[39]	1.5 μW		Quite low power harvesting
			[40]	1 μW		
			[41]	30 μW		
**Environment energy harvesting**	Solar energy		_	_		Not suitable for implanted devices
	Infrared radiation		[43]	4 mW	High power	Large dimension
	Wireless Harvest. energy	Ultrasonic	[48]	100 pW		Low power harvesting
			[49]	21.4 nW		
		Capacitive	_	_		For far range distance and having big size
		Inductive link	[57]	11 mW	High data rate and power transmission and batteries no needed	Limited carrier frequency due to tissue absorptions
			[72]	150 mW		
			[73]	10 mW		
			[74]	50 mW		
			[58]	22 mW		

The wireless harvesting energy using inductive coupling link is the best and most suitable method to power subcutaneous implanted devices with low band ISM frequencies. However, this method still suffers from several challenges. These issues and challenges may be overcome by considering certain parameters including coil dimensions, carrier frequency, modulation techniques and data transmission.

### Suitable inductive coupling links and coil shapes

In the wireless inductive coupling, the magnetic field is used to transfer data and power from the outer part to the inner part. In general, RF communications transmit a short-term low power irradiated from the reader antenna coil to provide fixed sinusoidal carrier amplitude that provides a stable power wireless transmission. The system consists of a vital organ of the integrated primary coil and is isolated outside the human body, which functions as a transmitter antenna, and the secondary coil is located inside the body, functioning as receiver. In most cases, they are tuned to the primary coil in series resonance to provide low impedance load for driving the transmitter coil. Meanwhile, the secondary coil is almost invariably in parallel. Thus, the best power transfer efficiency of inductive coupling link is achieved when both sides of the link are tuned at the same resonant frequency. In addition, the stability of the RF signals should provide high reading device implantation in terms of distances from the reader coil.

A number of studies have focused on the development of inductive links. The efficiency of the power transfer from such a system depends on many factors, such as coils, which depend on the quality factor of the coils and the coupling coefficient between two coils, geometries, shapes, size, alignment location, and core separated between coils. However, the transfer efficiencies at low frequencies are typically poor because of its narrow band and unfavorable conditions in most biomedical applications. For example, the implanted coil in neural recording is mounted subcutaneously with extremely limited headroom between the cortex and the skull [53-70,72-76]. High permeability cores typically used in a transformer to confine the magnetic flux are infeasible because of the strict size constraint and biocompatibility, which leads to weak mutual coupling. In addition, the achievable self-inductance of the coils is generally low when implemented by planar structure for low profile [65]. This two-coil coupling system typically suffers from poor power transfer efficiency, which drops sharply with distance because of its small size and low values.

The interest of researchers in resonance-based energy transfer has been ignited by the four-coil demonstration conducted by the MIT, which features high efficiency at midrange as opposed to the conventional two-coil resonant coupling [77-80]. The originally presented structure may not be suitable for biomedical implants because of its large size and bulkiness. A four-coil system specifically for biomedical implants was attempted in [81] with a 2.5 mm thick implanted coil, which may not be acceptable for implanted neural recording applications. Kiani *et al.*[82] proposed a three-coil link with high efficiency and power delivery. However, the tuning of the coupling between the secondary and load coils is not fiddling, and the high-density AC current induced by the high resonance in the secondary coil may damage living tissue. Furthermore, Litz wire implementation is not suitable for planar integration and batch production. Silay *et al.*[73] considered the effect of load resistance on the efficiency. However, they derived the optimum load from the conventional resonant coupling structure instead of the general inductive coupling structure and did not decouple the source impedance from the load. Thus, they claimed low achievable efficiency. Chen *et al.*[83] also studied this problem theoretically and presented a conceptual system.

In the near field, the distance between coils is smaller than the wavelength. Thus, coupling increases as the distance between coils decreases. Akin *et al.*[84] designed an implantable circular coil with a dimension of 5 mm × 8 mm × 2 mm and a carrier frequency of 4 MHz to offer a distance of 5 mm. Sauer *et al.*[57,67] used the same frequency presented in [84] to design external and implantable coils with outer dimensions of 50 mm and 20 mm, respectively, to offer a distance of 28 mm. For endoscopy monitoring, an implantable capsule with a coil dimension of 10 mm × 13 mm was designed by Lenaerts and Puers [72] to offer a distance of 205 mm. Harrison [67] designed a pancake coil with external coil (d_out_ = 52 mm, d_in_ = 10 mm) and secondary coil (d_out_ =10 mm, d_in_ = 5 mm) to offer a distance of 10 mm. Ahmadi and Jullien [85] designed an external spiral circular coil (d_out_ = 44 mm) and an implantable rectangular coil with dimension 15 mm to offer a distance of 40 mm. O’Driscoll *et al.*[86] produced a square inductive coupling operated with 915 MHz to produce a distance of 15 mm, the large size of this coil design is the issue.

**Figure 16:**
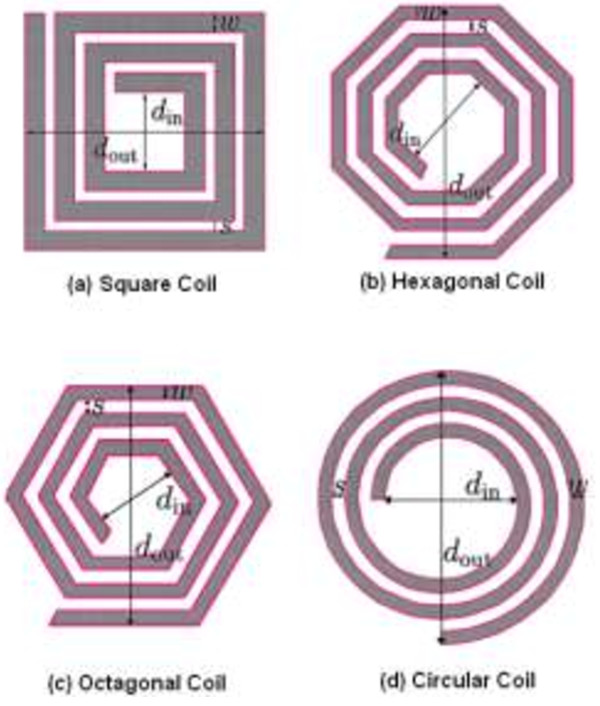
The conventional coils used in implanted devices.

Spiral rectangular coils with external and internal dimensions of 62 mm × 25 mm and 25 mm × 10 mm, respectively, and an operating frequency of 13.56 MHz were designed to offer a distance of 10 mm. Implantable coil size and short-range coupling are the issues [87]. Spiral square coils with dimensions of 70 mm × 8 mm and 20 mm × 8 mm were designed for an operating frequency of 1 MHz to 5 MHz. This design offers optimum coupling links in a distance of 10 mm, but the size and short range should still be considered [88]. Finally, circular coils with dimensions of 38 mm × 36 mm and 18 mm × 16 mm and an operating frequency of 742 KHz were designed to offer a distance of 1.5 mm. This design increases the printed board circuits and relatively occupies an area [89]. Figure 16 shows the conventional coils used in implantable devices, such as spiral polygon coil (spiral hexagon coil, spiral octagon coil), spiral square coil, and spiral circular coil [90]. Mutashar *et al.*[91] developed the spiral circular coil with dimensions of 56 mm × 10 mm and 11.6 mm × 5 mm, as well as an operating frequency of 13.56 MHz to offer a distance of 22 mm. Table 3 shows the bio-implantable devices exploiting inductive coupling links used in wireless power transmission.

**Table 3:** The bio-implanted inductive coupling links used for wireless power transmission

**Coil shape**	**Tx-Coil (mm)**	**Rx-Coil (mm)**	**Carrier frequency (MHz)**	**Distance (mm)**	**Modulation technique**	**Application**	**References**
**Circular**	-	(5 × 8 × 2)	4	5	Downlink-ASK Uplink-LSK	Neural recording system	[84] 1998
**Circular**	50	20	4	28	-	EEG signal detection	[57] 2005
**Tx-solenoid Rx-Cylinder**	d_in_ = 410	10 × 13	1	205	-	endoscopy	[72] 2006
	L = 300						
**Spiral pancake**	d_out_ = 52	d_out_ = 10	6.78	10	ASK	brine	[67] 2007
	d_in_ = 10	d_in_ = 5					
**Tx-spiral coil Rx-rectangular**	d_out_ = 44	4 × 8	13.56	40	LSK	Subcutaneous tissue	[85] 2009
**Square**	(2 × 2) cm^2^	(2 × 2) mm^2^	915	15	_		[86] 2009
**Spiral rectangular**	62 × 25	25 × 10	13.56	10	ASK	Implanted micro-system	[87] 2011
**Spiral square**	70 × 8	20 × 8	1-5	10	ASK		[88] 2012
**Circular**	d_out_ = 38	d_out_ = 18	742 KHz	10	_	Neural recording system	[89] 2012
	d_in_ = 36	d_in_ = 16					
**Spiral circular**	d_out_ = 56	d_out_ = 11.6	13.56	22	ASK	Nerves and muscles stimulator	[91] 2014
	d_in_ = 10	d_in_ = 5					

### Suitable carrier frequency for implanted devices

**Figure 17:**
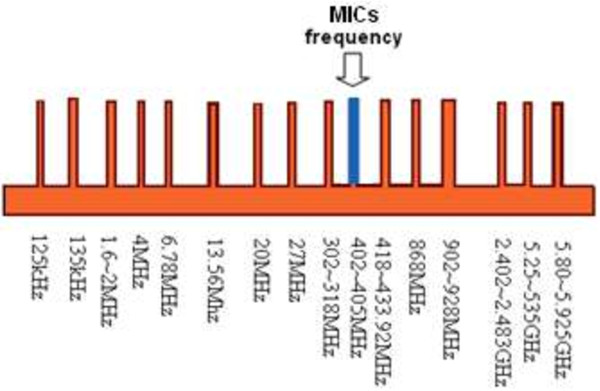
The ISM and MICs frequency bands.

Carrier frequency is significant in designing implantable devices and biotelemetry systems. Most implantable devices are powered by low frequencies (>1 MHz). However, the standard safety level with respect to human body exposure to RF electromagnetic fields ranges from 3 kHz to 30 GHz [92]. The standard frequencies according to the Medical Implants Communication Service (MICS) range from 402 MHz to 405 MHz, which involves a number of allowable frequencies such as 27 MHz. The second standard is the ISM standard dealing with 125 kHz to 135 kHz, 6.78 MHz, 13.56 MHz, 27.125 MHz and 40.68 MHz, as well as 433.92 MHz, 869 MHz, and 2.4 GHz in an ultra-high frequency band. The frequency range of 3 kHz to 30 MHz is widely used in transcutaneous wireless because of its ability penetrate water and skin over a short range and cannot heat the surrounding biological tissue. Figure 17 shows the ISM and MICS frequency bands used for medical applications.

### Suitable modulation transmission technologies

Digital modulation impressed the digital signal onto a carrier signal for data transfer. However, digital modulation has drawbacks related to difficulty in designing complex structures, analog counterparts, and bandwidth size. The major criteria for selecting the type of modulation schemes are based on the application, simplicity, system efficiencies, power, and bandwidth [93,94]. The common modulation techniques used in implantable devices are amplitude shift keying (ASK), frequency shift keying, and phase shift keying. Hannan *et al.*[71] analyzed these techniques and concluded that ASK modulation is the most suitable technique that can be used in implantable biomedical devices.

### Low data rate transmission

The required data rate between the two parts of the inductive link varies depending on the application. Retinal implants, cochlear implants, and endoscopy capsules require high data rate transmission. The stimulator to be implanted also depends on the number of electrodes. Inductive coupling link is the best and most suitable method to power subcutaneous implantable devices because of its lower band ISM frequencies and higher data rate than the other methods.

### Low cost and commercialization

In the 1950s, when the first biomedical implantable devices were implanted, the focus was on scientific success, and the economic aspects were not important at that time. However, with the increasing use of the biomedical implantable devices, the economy of the implantable devices is becoming very important issue. In addition to safety and comfort for the patient. Thus, low-cost and simple designs became an important factor and challenges for the designers [9]. Now days, one of the most important factors influencing the design and manufacture of the devices planted inside the body is the commercial factor. Most of the companies, which manufacturer the biomedical implantable devices lay in their accounts cost business process, commercial commercialization and the scientific commercialization [95].

The global market for implanted medical devices is significant and growing and has become a lucrative business and vitality because of its relationship to the human health. For example, over 600,000 pacemakers were implanted worldwide in 2003, with 3 million of the devices in use at that time. In 2004, the overall market for cardiac rhythm management was estimated to be $8.9 billion, and by 2007 the total market for implantable and ingestible devices was predicted to exceed $24.4 billion. In addition to pacemakers and defibrillators, implantable devices now include pumps for diabetes and pain management, neurostimulators for pain therapy, and devices similar to pacemakers to electrically stimulate the stomach, throat, and other muscles.

Regarding to the methods given in this study, the Implantable biomedical devices is classified into two types. The first type includes devices powered by energy harvested from the human body. The second method includes those powered by energy harvested from the environment, and for commercial purposes rating; it has been divided according to the commercial commercialization and scientific commercialization and the possibility of marketing. No days, the implanted devices which used the human energy harvesting such as piezoelectric generator is one of the best methods in which manufacturers prefer, because of their low-cost and the increasing demand. However, for scientific point of marketing, we believe that the inductive coupling method is better scientifically, and currently it is within the manufacturer’s plans and continuous investigations to be more concentration for commercial marketing.

## Conclusion

This study describes the various energy harvesting techniques used in implanted biomedical devices. All methods for harvesting energy from environmental sources and human body motion and vibration are reviewed and discussed. These methods can be used in portable devices and implantable devices, such as implantable micro-systems, cochlear implant, and pacemakers. The major characteristics of harvesting energy by human body motion involves kinetic and thermoelectricity generators. Kinetic harvesting includes piezoelectric material, electrostatic generators, and magnetic induction generator. Environment energy harvesting was divided as shown in Figure 1. The characteristics of physical–mathematical methods of energy harvesting are detailed, including the advantages and disadvantages of each method. Overall, after comparing the past evolution of all methods, including piezoelectric, electrostatic, electromagnetic, thermo-electric, batteries, fuel cells, ultrasound, and inductive coupling link, we concluded that the inductive link remains the best mode of harvesting energy. Electronic technology is expected to continue its evolution of decreasing energy consumption to develop energy harvesting methods. In the future, implantable and portable medical devices are expected to be free of batteries.

## Abbreviations

MIT: Massachusetts institute of technology; MEMS: Micro-electromechanical systems; ISM: Industrial, scientific, and medical; MICS: Medical implants communication service; SP: Serial-to-parallel topology; PS: Parallel-to-serial topology; SS: Serial-to-serial topology; PP: Parallel-to-parallel topology; ESG: Electrostatic generator; Q: Quality factor; K: Coupling coefficient; EEH: Environmental bio-energy harvesting.

## Competing interests

The authors declare that they have no competing interests.

## Authors’ contributions

SM: Collection, organizing, modification, revision, review of the literature and preparing the manuscript. MAH: Modification, editing and preparing the manuscript. All the authors read and approved the final manuscript.
